# Intratumoral macrophages contribute to epithelial-mesenchymal transition in solid tumors

**DOI:** 10.1186/1471-2407-12-35

**Published:** 2012-01-24

**Authors:** Anne-Katrine Bonde, Verena Tischler, Sushil Kumar, Alex Soltermann, Reto A Schwendener

**Affiliations:** 1 Institute of Molecular Cancer Research, University of Zürich, Winterthurerstrasse 190, CH-8057 Zürich, Switzerland; 2 Institute of Surgical Pathology, University Hospital Zürich, Schmelzbergstrasse 12, CH-8091 Zürich, Switzerland

**Keywords:** Tumor-associated macrophages (TAMs), Macrophage depletion, Clodronate liposomes, Tumor progression, Tumor invasion, Epithelial-mesenchymal transition (EMT), TGF-β

## Abstract

**Background:**

Several stromal cell subtypes including macrophages contribute to tumor progression by inducing epithelial-mesenchymal transition (EMT) at the invasive front, a mechanism also linked to metastasis. Tumor associated macrophages (TAM) reside mainly at the invasive front but they also infiltrate tumors and in this process they mainly assume a tumor promoting phenotype. In this study, we asked if TAMs also regulate EMT intratumorally. We found that TAMs through TGF-β signaling and activation of the β-catenin pathway can induce EMT in intratumoral cancer cells.

**Methods:**

We depleted macrophages in F9-teratocarcinoma bearing mice using clodronate-liposomes and analyzed the tumors for correlations between gene and protein expression of EMT-associated and macrophage markers. The functional relationship between TAMs and EMT was characterized *in vitro *in the murine F9 and mammary gland NMuMG cells, using a conditioned medium culture approach. The clinical relevance of our findings was evaluated on a tissue microarray cohort representing 491 patients with non-small cell lung cancer (NSCLC).

**Results:**

Gene expression analysis of F9-teratocarcinomas revealed a positive correlation between TAM-densities and mesenchymal marker expression. Moreover, immunohistochemistry showed that TAMs cluster with EMT phenotype cells in the tumors. *In vitro*, long term exposure of F9-and NMuMG-cells to macrophage-conditioned medium led to decreased expression of the epithelial adhesion protein E-cadherin, activation of the EMT-mediating β-catenin pathway, increased expression of mesenchymal markers and an invasive phenotype. In a candidate based screen, macrophage-derived TGF-β was identified as the main inducer of this EMT-associated phenotype. Lastly, immunohistochemical analysis of NSCLC patient samples identified a positive correlation between intratumoral macrophage densities, EMT markers, intraepithelial TGF-β levels and tumor grade.

**Conclusions:**

Data presented here identify a novel role for macrophages in EMT-promoted tumor progression. The observation that TAMs cluster with intra-epithelial fibroblastoid cells suggests that the role of macrophages in tumor-EMT extends beyond the invasive front. As macrophage infiltration and pronounced EMT tumor phenotype correlate with increased grade in NSCLC patients, we propose that TAMs also promote tumor progression by inducing EMT locally in tumors.

## Background

The malignant potential of solid tumors highly depends on adjacent stromal cells such as cancer associated fibroblasts (CAFs), mesenchymal stem cells (MSCs) and immune cells [1-4]. Macrophages belong to the latter, and their migration from the stroma into tumors correlates inversely with patient survival in many cancers, among others breast, lung and thyroid carcinoma as well as Hodgkin's lymphoma [5-9]. These correlations have largely been related to the macrophage secretome which involves factors that stimulate tumor cell proliferation and survival, angiogenesis and release of proteases essential for extracellular matrix (ECM) remodeling [10-12]. Vice versa, several paracrine signaling loops have been identified through which macrophages orchestrate invasion of tumor epithelia into its own newly formed desmoplastic stroma [13-18].

An important step in tumor progression is the acquisition of invasive properties by tumor cells. EMT is a well characterized mechanism, through which epithelial cells trans-differentiate and acquire an invasive mesenchymal phenotype [19,20]. EMT has recently been recognized for its involvement in disease progression and the mechanisms have been linked to metastasis and to the generation of cancer stem cell-like cells [21-25]. Concordantly, we have previously identified strong correlations between EMT-associated marker expression in non-small cell lung cancer (NSCLC) patients and various clinico-pathologic parameters of tumor progression, such as size and decreased survival [26].

As EMT represents a crucial step in disease progression it is of importance to identify and characterize the mechanisms regulating this process. Whereas it is well established that the stroma hosts cytokines, growth factors and enzymes that can induce EMT, the sources of these factors remain to be fully indentified [27-36]. CAFs, MSCs and Th2 polarized CD4^+^/CD8^+ ^T-lymphocytes have all been shown to contribute to EMT at the tumor-stroma interface [37-41]. Pro inflammatory macrophages (classically activated or M1) have likewise been shown to induce EMT at the invasive front mainly through TNF-α mediated stabilization of Snail, a key mediator and marker of EMT [21,42]. Interestingly, M1 TAM induced EMT in tumor cells located at the invasive front correlates with metastatic disease in a murine breast cancer model which underscores the importance of both EMT and macrophages in disease progression [21].

In this study we asked if TAMs regulate EMT in intratumoral epithelial cells, as well at the invasive front. We used clodronate-liposomes (clodrolip) to deplete TAMs in F9-teratocarinomas. In combination with established *in vitro *culture techniques we identified alternatively activated M2 macrophages as potent regulators of EMT in F9-tumor cells as well as in the murine epithelial mammary gland cell line, NMuMG. In a candidate screen, we identified macrophage-derived TGF-β and consecutive activation of the β-catenin pathway as mechanisms of action. An important aspect of this study was to evaluate the clinical relevance of TAM-induced EMT in disease progression. This was addressed in a NSCLC tissue microarray cohort, which confirmed a significant correlation between intratumoral macrophage density, EMT markers, intraepithelial TGF-β levels and tumor grade. The data presented here identify intratumoral macrophages as potent regulators of intraepithelial EMT, adding another level to the importance of macrophages in EMT and disease progression.

## Methods

### Antibodies and reagents

Primary antibodies: Anti-rat-E-cadherin, anti-rabbit-β-actin, anti-mouse-vimentin, anti-rabbit-fibronectin all from Abcam, Cambrigde, MA; anti-rabbit-β-catenin (Sigma-Aldrich, St. Louis, MO), CD68-Alexa-488 and F4/80-Alexa-647 (AbD Serotec, Düsseldorf, Germany) and anti-mouse-active-β-catenin (Millipore, Billerica, MA). Secondary antibodies: Goat-anti-rat-IgG-TRITC (Sigma-Aldrich, St. Louis, MO); chicken-anti-rabbit-Alexa-594 (Molecular Probes, Carlsbad, CA); biotin-SP-donkey-anti-rabbit-IgG, biotin-SP-donkey-anti-rat-IgG, and biotin-SP-donkey-anti-mouse-IgG (Jackson ImmunoResearch Laboratories, INC, Suffolk, UK). Streptavidin-HRP (Biolegends, San Diego, CA) was used to detect biotin labeled secondary antibodies. Recombinant TGF-β1 and TGF-β neutralizing antibody was purchased from R&D Biosystems (Minneapolis, MN) and recombinant EGF was kindly provided by Dr. A. Mueller, IMCR, University of Zürich, Switzerland. LEAF (low endotoxin azide free) purified mouse IgG_1_, κ isotype control antibody was obtained from BioLegends (San Diego, CA). Recombinant IL-4 and IL-13 were from Biosource (Camarillo, CA).

### Cell lines and conditioned media

F9-teratocarcinoma cells (ATCC CRL-1720) were grown on 0.01% gelatin. NMuMG-cells (CRL-1636) were kindly provided by Prof. G. Christofori, Center for Biomedicine, University of Basel, Switzerland. Both cell lines were cultured at 37°C, 5% CO_2 _in DMEM/10% FBS/0.8% penicillin-streptomycin. RAW264.7 macrophages (Sigma-Aldrich, St. Louis, MO) were cultured at 37°C, 5% CO_2 _in RPMI1640/10% FBS/0.8% penicillin-streptomycin/1% Na-pyruvate (GIBCO, Basel, Switzerland). Conditioned medium was generated by culturing cells at 80% confluence in DMEM/10% FBS/0.8% penicillin-streptomycin for 24 h followed by sterile filtration. RAW264.7 macrophages were M2 polarized by culturing in DMEM/10% FBS plus recombinant IL-4 and IL-13 for 48 h (each 10 ng/ml) as previously described [43].

### F9 tumors and macrophage depletion

F9-tumors were generated in female SV129S1 mice (Charles River, Sulzfeld, Germany) and liposomes were prepared as previously described [11]. Mice were kept in standard housing and normal diet at the animal facility of the University of Zürich. Animal studies were approved by the Veterinary Department of the Canton Zürich and performed under license 183/2006 issued to R.A. Schwendener. The control group (n = 6) received empty liposomes (100 μl/20 g body weight, i.p.), the test group (n = 6) clodrolip (1.5 mg clodronate/20 g body weight, i.p.) starting 6 h post tumor inoculation and followed by the same dosage every 3rd day for 20 days. Tumors subjected to immunohistochemistry and protein analysis were stored in Hanks salt buffer (GIBCO, Basel, Switzerland) at -80°C. Tumors subjected to q-PCR were stored in RNAlater as described by the provider (Qiagen, Valencia, CA). Data are shown from two independent experiments and non-responders as assayed by q-PCR of *Csfr-1 *were excluded from the study unless otherwise noted.

### H&E staining, immunohistochemistry and quantification of frozen F9 tissue sections

Frozen sections (8 μm) were acetone fixed. The sections were either stained with haematoxylin and eosin (DAKO, Glostrup, Denmark) following the providers protocol or blocked with 1% BSA/TBS prior to immunostaining. For immunohistochemistry, the sections were incubated with primary and secondary antibodies overnight at 4°C. Nuclei were stained with DAPI (1 μg/ml). The sections were mounted with Vectashield (Vector Labs, Burlingame, CA) and visualized with an Olympus fluorescence microscope (1X81) using the CellR software (Olympus, Hamburg, Germany). The pictures were merged in Adobe Photoshop CS4. Macrophage density was quantitatively estimated by counting the absolute number of CD68^+ ^and F4/80^+ ^cells and the total number of DAPI positive cells in defined areas throughout tumor sections using ImageJ software (NIH, Bethesda, MD). Quantification and correlation of macrophage density and tumor cell expression of EMT-associated markers was similarly done in a quantitative manner, using ImageJ to count the absolute number of CD68^+ ^and EMT-marker positive cells in defined areas throughout the tumor sections. The quantifications were done on 6-10 individual sections from various control and clodrolip treated tumors sampled from two independent experiments.

### In vitro induction of EMT and immunofluorescence analysis of F9-and NMuMG-cells

F9-and NMuMG-cells were cultured on sterile glass coverslips in F9-CM, N-CM, M-CM +/- LEAF purified IgG_1 _control antibody (1 μg/ml) or TGF-β neutralizing antibody (1 μg/ml), DMEM/10% FBS +/- rEGF (50-100 ng/ml) or +/- rTGF-β1 (2 ng/ml). The medium was renewed every 24 h. The cells were harvested at the time points annotated, fixed with 3% formaldehyde, stained and visualized as described for frozen sections.

### In vitro invasion assay

The cells were starved in serum free medium for 6 h and seeded (100.000 cells/well) in Boyden chambers (Corning, NY, 8 μm pore size) coated with 50 μl 1% Matrigel (BD Biosciences, Rockville, MD). F9-CM, N-CM and M-CM +/-LEAF purified IgG_1 _control antibody (1 μg/ml) or +/- TGF-β neutralizing antibody (1 μg/ml) were used as chemoattractants. The assay was incubated for 48 h at 37°C, 5% CO_2_. The relative number of invading cells was estimated by resazurin live cell detection using the provider's protocol (Invitrogen, Carlsbad, CA). Fold invasion was calculated relative to control conditions.

### TOPFLASH reporter assay

The TOPFLASH reporter assay was established as previously described [44]. The fold values were calculated as TOPFLASH/FOPFLASH, where TOPFLASH is the plasmid expressing luciferase downstream of three wild type β-catenin/Tcf binding sites, and FOPFLASH is the plasmid with mutated binding sites. Renilla pRL SV40 was included as transfection control. Luciferase was detected using the Dual Glo Luciferase detection kit (Promega, Madison, WI). The cells were transfected two days prior to luciferase readout using a standard in-house transfection protocol. Fold changes were calculated relative to controls.

### Western blots

F9-and NMuMG-cells were lysed 1% NP-40, 100 mM orthovanadate, 100 mM 3-indoleacetic acid (IAA), 100 mM phenylmethylsulfonylfluoride (PMSF) at annotated time points and snap frozen in liquid nitrogen. Frozen tumors were cut into small pieces and soaked in lysis buffer and homogenized using an Ultra Turrax T8 homogenizer (IKA-Werke, Staufen, Germany). Protein concentration was determined by Bradford analysis (Bio-Rad, Reinach BL, Switzerland). The blots were quantified using ImageQuant 5.2 software (Amersham Biosciences, Piscataway, NJ). Protein expression was normalized to β-actin levels.

### Quantitative real time PCR

Total RNA was isolated from homogenized F9-tumors using the RNAeasy kit (Qiagen, Valencia, CA). cDNA was synthesized using the Omniscript reverse transcriptase kit (Qiagen, Valencia, CA). Q-PCRs were carried out using the LightCycler 480 instrument (Roche Diagnostics, Rotkreuz, Switzerland). PCR program: 95°C, 5 min, 45 cycles of 10 s 95°C, 25 s annealing and 15 s 72°C. Primers were obtained from Microsynth, Switzerland, (for primer sequences and annealing temperatures, see Additional file 1: Table S1). The quality of the PCR-products was assayed on 1.5% agarose gels. Expression of all target genes was normalized to β-actin and GAPDH. All samples were run in duplicates; n = 5-6/per group. Fold change was calculated as clodrolip treated versus control tumors using the Pfaffl equation [45].

### NSCLC tissue microarrays and patient cohort

The selection of NSCLC patient tissue samples and manufacture of the tissue microarrays (TMAs) were done as previously described [26]. In brief, formalin-fixed and paraffin-embedded tumor tissues of 532 NSCLC patients were reviewed by two pathologists and two representative tissue cores (0.6 mm) were assembled into 3 TMAs. Patients having obtained neo-adjuvant chemotherapy were excluded. Sarcomatoid carcinomas were excluded from this study and EMT was strictly defined by expression of EMT-associated protein markers and not by morphology (n final = 491). The study was approved by the institutional review board of the University Hospital Zürich under reference number StV-29-2009.

### NSCLC immunohistochemistry and interpretation

Immunohistochemistry on 4 μm sections from the TMA blocks was performed using automated immunohistochemistry platforms from either Ventana (Ventana Medical Systems, Tucson, AZ) or Bond (Vision Biosystems, Melbourne, Australia). Following primary monoclonal antibodies were used: anti-CD68 (DAKO-Cytomation, clone PG-M1, 1:50 dilution, Glostrup, Denmark), anti-E-cadherin (Cell Marque, clone EP700Y, 1:200), anti-β-catenin (BD Transduction laboratories, clone 14, 1:50, Lexington, KY), anti-vimentin (DAKO-Cytomation, 1:250), Pab anti-periostin (BioVendor, 1:500, Modrice, Czech Republic) and anti-TGF-β1 (Santa Cruz, 1:100 dilution). Detection was done using the UltraVIEW-DAB (Ventana Medical Systems) or the Refine-DAB (Bond) detection kits, including respective secondary antibodies. Distinct intra-epithelial CD68^+ ^macrophage density was quantitatively scored by AKB and by two pathologists (VT and AS) on a multi-headed microscope (Zeiss Axioscope 2 MOT) using a four-tiered system: 0 (negative), 1+ (few to some CD68^+ ^macrophages), 2+ (moderate number of CD68^+ ^macrophages), and 3+ (multiple CD68^+ ^macrophages). Membranous β-catenin (AKB and VT) and membranous E-cadherin (VT and AS) were evaluated for staining intensity according to a four-tiered system: 0 (negative, no detectable staining), 1+ (weak, faint discontinuous membrane staining), 2+ (moderate and continuous membrane staining), 3+ (strong and continuous membrane staining). Cytoplasmic β-catenin (AKB and VT), cytoplasmic vimentin (AKB and AS), cytoplasmic periostin (VT and AS) were scored due to staining intensity: 0 (negative), 1+ (weak), 2+ (moderate), and 3+ (strong) and TGF-β1 (AKB and VT) was scored for intraepithelial staining intensity: 0 (negative), 1+ (weak), 2+ (moderate), and 3+ (strong).

### Statistical methods and correlation interpretation

The statistical analyses of all *in vitro *assays were performed using the GraphPad Prism 5 software (GraphPad Software, La Jolla, CA). All data are reported as mean ± SEM. The significance levels were evaluated by two tailed, unpaired t-tests; *P < 0.05, **P < 0.01. The statistical and correlation analyses of the NSCLC tissue samples were done in SPSS 16.0 for windows (IBM, Somers, NY) using the Spearmann correlation coefficient as a readout for degree of correlation with *P < 0.05.

## Results

### Depletion of TAMs reduces mesenchymal gene expression in F9-terato-carcinomas

**Figure 1:**
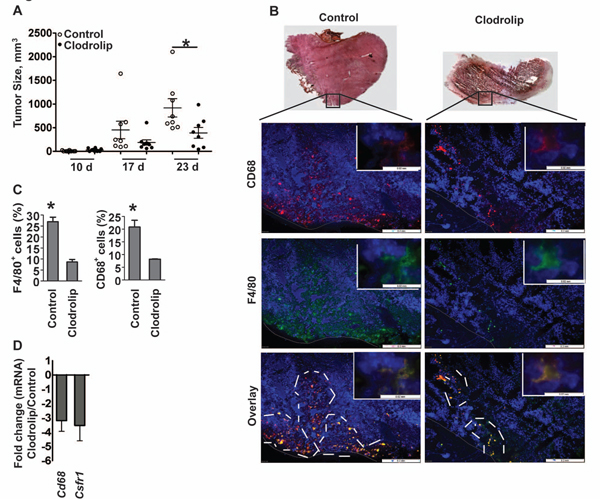
**Clodrolip depletes macrophages in F9-teratocarcinoma bearing mice**. (**A**) Size distribution of control and clodrolip tumors at day 10, 17 and 23 after treatment initiation. (**B**) Upper panel: HE staining of a control tumor section (left) and a clodrolip treated tumor section (right), magnification 2.5×. Note the large necrotic center in the clodrolip treated tumor (right panel). Panels: Immunohistochemical analysis of CD68^+ ^(red) and F4/80^+ ^(green) density in control (left) and clodrolip treated (right) tumors, magnification 10×. Overlay of double-positive macrophages is shown in orange (lower panel); scale bar = 0.2 mm. Macrophage-rich regions are delineated by white lines. Macrophage magnification of 100 × is displayed in each picture at the upper right corner; scale bar = 0.02 mm. Nuclei were stained with DAPI (blue). (**C**) Quantitative analysis of F4/80^+ ^(left) and CD68^+ ^(right) cell density in control and clodrolip treated tumors; n = 3/group, bars: means ± SEM; *P < 0.05, unpaired *t*-test. (**D**) Relative gene expression (mRNA levels) of *Cd68 *and *Csfr-1 *in clodrolip treated vs. control tumors; n = 6, bars: means ± SEM.

**Figure 2:**
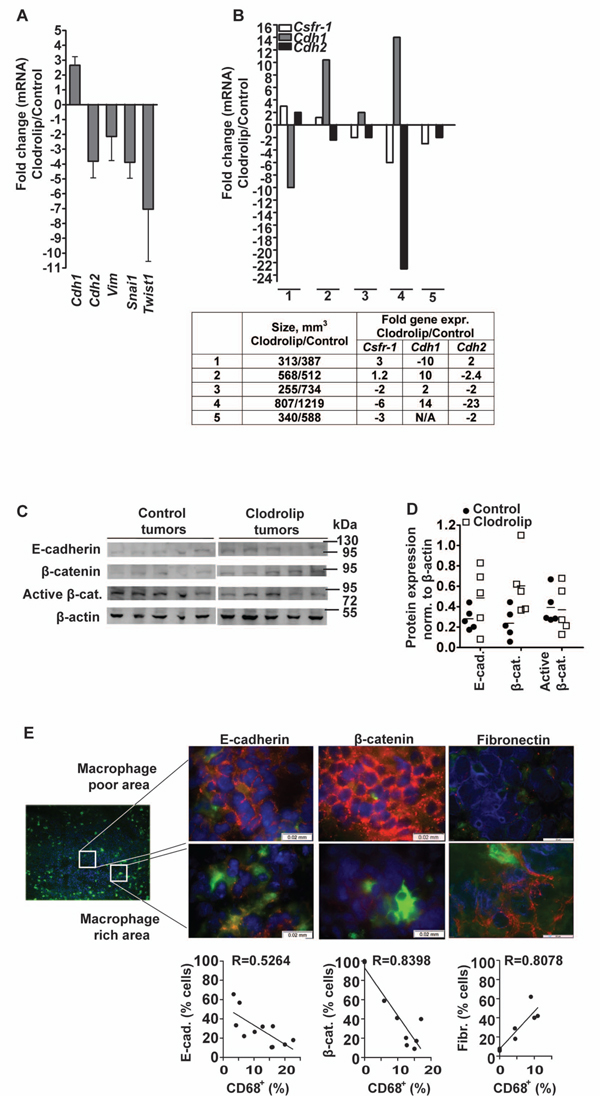
**Macrophage depletion correlates with reduced mesenchymal marker expression in F9-teratocarcinomas**. (**A**) Relative gene expression (mRNA levels) of *E-cadherin *(*cdh1*), *N-cadherin *(*cdh2*), *vimentin *(*vim*), *Snail *and *Twist *in clodrolip treated vs. control tumors; n = 5-6, bars: means ± SEM. The tumors analyzed in this experiment were collected from two independent depletion experiments. Non-responders were excluded. (**B**) Relative gene expression (mRNA levels) of *Csfr-1, E-cadherin *(*cdh1*) and *N-cadherin *(*cdh2*) in size matched clodrolip treated vs. control tumors; n = 5-6, bars: means ± SEM. The tumors analyzed in this figure were collected from a single depletion experiment. The table shows individual volumes and gene expression levels of paired treated vs. control tumors. (**C**) Western blot analysis of E-cadherin, total β-catenin and active β-catenin expression in control and clodrolip treated tumors; n = 5. (**D**) Quantification of the western blot is shown in Figure C. (**E**) Immunofluorescence analysis of CD68^+ ^macrophages (green), E-cadherin (red, left panel), β-catenin (red, mid panel) and fibronectin (red, right panel) in a macrophage poor area (upper panel, magnification 100×) and in a macrophage rich area (lower panel, magnification 100×) in a control tumor section (left picture, magnification 10×). The nuclei were stained with DAPI (blue). Scale bar = 0.02 mm. The graphs indicate correlations between CD68^+ ^density and expression of the annotated EMT markers. Each data point represents analysis of one tumor section; n = 3-5/group. The linear regression coefficient R is indicated.

We used clodrolip to deplete macrophages in F9-teratocarcinoma tumors inoculated subcutaneously in Sv129J mice. In accordance with previous reports [11] we observed a significant reduction in tumor growth in response to treatment over the course of three weeks (Figure 1A). H&E tissue staining revealed large regions of necrosis in clodrolip treated tumors. Reduction in tumor size correlated with a 70-80% decrease in F4/80^+ ^and CD68^+ ^macrophage density (Figure 1B and 1C). Finally, macrophage depletion was confirmed by gene expression analysis of *CD68 *and colony stimulating factor-1 receptor *Csfr-1 *(Figure 1D). The tumors were next evaluated for gene expression of key EMT-markers (Figure 2A). The tumors evaluated in this study were selected from two independent depletion experiments. Non-responders were identified by immunohistochemistry and RT-PCR analysis of *Csfr-1 *and *CD68 *expression and excluded from the evaluation. The analysis revealed a relative reduction in expression of two mesenchymal markers *vimentin *(*Vim*, ~2.5-fold) and *N-cadherin *(*Cdh2*, ~4-fold), whereas expression of the epithelial marker *E-cadherin *(*Cdh1*) was increased ~3-fold in clodrolip treated tumors relative to controls. Expression of the EMT-associated transcription regulators *snail (Snai1) *and *twist (Twist1)*, both of which repress *E-cadherin *transcription [46,47] were decreased in clodrolip treated tumors ~4-fold and ~7-fold, respectively (Figure 2A). A hallmark of EMT is the E-cadherin/N-cadherin switch [48]. To asses if macrophage depletion correlated with a shift in cadherin expression in individual tumors, five tumors from a single depletion experiment were size matched and analyzed by RT-PCR (Figure 2B). Of the five clodrolip treated tumors 2 were identified as non-responders, whereas the remaining three displayed 2-6 fold depletion of macrophages relative to their size matched control tumors. The relative macrophage density, as assessed by *Csfr-1 *gene expression levels, correlated strongly with inverse *Cdh1 *and *Cdh2 *expression which confirms the correlation between macrophage density and the cadherin switch in the individual sets of tumors (clodrolip/control). Western blotting confirmed a relatively high expression level of E-cadherin as well as its cytoplasmic interaction partner β-catenin in three of five clodrolip treated tumors (Figure 2C). The β-catenin pathway can mediate EMT when transcriptionally active complexes with TCF/LEF are formed upon dissociation from the membranous E-cadherin [34,44,49]. Thus, we examined the levels of active β-catenin in the two tumor groups using an antibody specifically detecting the active form of β-catenin dephosphorylated on Ser37/Thr41. Quantification of the western blot revealed no obvious difference in the levels of active β-catenin between the two tumor groups (Figure 2D) suggesting that the levels of activated β-catenin in total tumor tissues were independent of clodrolip treatment.

Macrophages are known to infiltrate and locate in clusters rather to being distributed evenly throughout the tumor tissue. Thus, the tumors were analyzed by immunohistochemistry for intra-tumoral macrophage density and expression of EMT markers in adjacent tumor cells (Figure 2E). This analysis identified local correlations between tumor cell expression of EMT markers and intratumoral CD68^+ ^macrophage density. Whereas E-cadherin and β-catenin localized to the plasma membrane of cells in areas with low CD68^+ ^macrophage densities (Figure 2E, upper panel), expression of both proteins was compromised and partially lost in areas with high CD68^+ ^densities (Figure 2E, lower panel). Conversely, fibronectin expression was increased in areas with high CD68^+ ^densities. Thus, intratumoral TAM density correlated locally with a mesenchymal phenotype in F9-tumors.

### M2 macrophages induce EMT and stimulate the invasive properties of F9-and NMuMG cells in vitro

**Figure 3:**
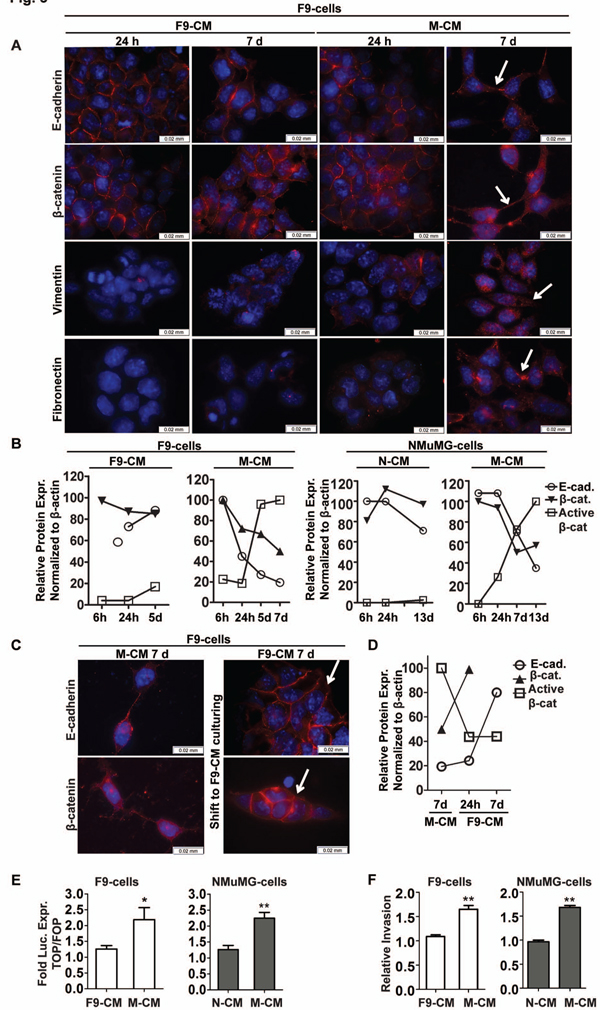
**M2 polarized macrophages induce trans-differentiation, activation of the β-catenin pathway and invasion of F9- and NMuMG cells *in vitro***. (**A**) Immunofluorescence of E-cadherin, β-catenin, vimentin and fibronectin expression and cellular localization in F9-cells cultured in F9-CM or M-CM for 24 h or 7d. Pictures of NMuMG-cells are shown in Additional file 2: Figure S1. Scale bar = 0.02 mm. Proteins are colored red and nuclei were stained with DAPI (blue). Arrows indicate the location of the annotated protein in cells cultured in M-CM for 7d. (**B**) Relative protein expression normalized to β-actin levels (quantified from western blots shown in Additional file 2: Figure S2.) of E-cadherin, total and active β-catenin in F9-cells (left panels) and NMuMG-cells (right panels) cultured in F9-CM, N-CM or M-CM for 6 h, 24 h, 5d, 7d, and 13d. (**C**) Immunofluorescence of E-cadherin and β-catenin expression and cellular localization in F9-cells cultured in M-CM for 7d (left panel), followed by additional culture in F9-CM for 7d (right panel). Scale bar = 0.02 mm. Proteins are colored red and nuclei were stained with DAPI (blue). Arrows indicate the location of the annotated protein in cells after 7d culture in F9-CM. (**D**) Relative protein expression normalized to β-actin levels (quantified from western blot shown in Additional file 2: Figure S2A) of E-cadherin, total and active β-catenin in F9-cells cultured in M-CM for 7d, followed by F9-CM for additional 24 h and 7d. (**E**) Fold luciferase expression (TOPFLASH/FOPFLASH) in F9-cells (left) and NMuMG-cells (right) after 7d or 13d of culture, respectively, in F9-CM, N-CM or M-CM; n = 7; bar = means ± SEM; *P < 0.05. **P < 0.005, unpaired *t*-test. (**F**) Invasion by F9-cells (n = 8) and NMuMG-cells (n = 3) in response to F9-CM, N-CM or M-CM (48 h, 1% Matrigel) calculated relative to F9-CM or N-CM, respectively. **P < 0.005, unpaired *t*-test; bars = means ± SEM.

The functional relationship between M2 macrophages and induction of tumor cell EMT was further investigated in 2D culture systems. To this end, we pre-stimulated murine RAW264.7 macrophages for 48 h with IL-4 and IL13 to polarize them to a M2 phenotype [43]. F9-teratocarcinoma [50] and NMuMG-cells [29-32] were cultured in conditioned medium generated either by F9-cells (F9-CM), NMuMG-cells (N-CM), or by M2 polarized RAW 264.7 macrophages (M-CM). The cells were evaluated for morphology and cellular localization of E-cadherin, β-catenin, vimentin and fibronectin (Figure 3A, Additional file 2: Figure S1 and data not shown). Both cell lines displayed an epithelial morphology when cultured in F9-CM or N-CM and E-cadherin and β-catenin localized to the cell membrane. A similar profile was observed in cells cultured for 24 h in M-CM. However, after long term culture in M-CM (7 days for F9-cells, 13 days for NMuMG-cells) the cells acquired a mesenchymal-like morphology (Figure 3A right panel, Additional file 2: Figure S1, right panel). This change in morphology correlated with reduced expression of E-cadherin and β-catenin and an increase in active β-catenin levels (Figure 3B, Additional file 2: Figure S2). Moreover, the immunofluorescence analysis revealed a time dependent increase in expression of two mesenchymal markers, vimentin and fibronectin (Figure 3A, Additional file 2: Figure S1). This M-CM induced mesenchymal phenotype was reversible in F9-cells in a time dependent manner (Figure 3C and 3D, Additional file 2: Figure S2A). Upon removal of M-CM followed by culturing in F9-CM for 7 days, E-cadherin and β-catenin expression levels gradually increased and the proteins re-localized to the cell membrane, while the level of active β-catenin gradually decreased,.

We next tested if β-catenin was transcriptionally activated upon long term M-CM culturing. For this purpose we used the TOPFLASH/FOPFLASH reporter assay [44]. M-CM culturing led to an approximately 2-fold increase in β-catenin dependent luciferase activity in both cell lines, confirming that β-catenin became transcriptionally activated upon M-CM treatment (Figure 3E).

A widely accepted physiological consequence of EMT is tumor cell invasion [19,22-24,31,33]. Thus, the invasive properties of F9-and NMuMG-cells were assessed *in vitro *using Matrigel coated transwells. Both cell lines were slowly invading under non-stimulated conditions and their invasive properties significantly increased in response to M-CM (Figure 3F). Collectively, the data show that M-CM generated by M2 polarized macrophages induces mesenchymal trans-differentiation, activates the β-catenin pathway and increases the invasive properties of both cell lines.

### TGF-β signaling is crucial for M2 macrophage induced tumor cell EMT

**Figure 4:**
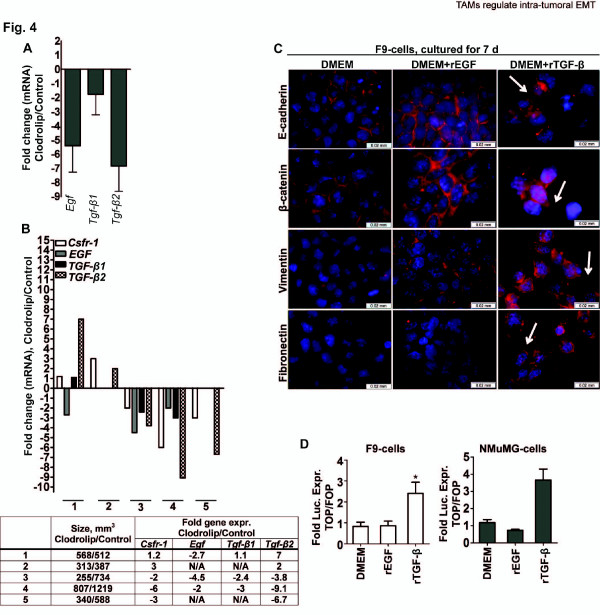
**TGF-β induces trans-differentiation in F9-and NMuMG-cells *in vitro***. (**A**) Relative gene expression (mRNA levels) of *Egf*, *Tgf-β1 *and *Tgf-β2 *in clodrolip treated vs. control tumors; n = 5-6, bars: means ± SEM. (**B**) Relative gene expression (mRNA levels) of *Csfr-1, Egf, Tgf-β1, and Tgf-β2 *in size matched clodrolip treated vs. control tumors; n = 5-6, bars: means ± SEM. The table shows individual volumes and gene expression levels of paired treated vs. control tumors. The tumors analyzed in this figure were collected from a single depletion experiment. (**C**) Immunofluorescence of E-cadherin, β-catenin, vimentin and fibronectin expression and cellular localization in F9-cells cultured in DMEM +/-EGF (50 ng/ml) and +/- TGF-β1 (2 ng/ml) for 7d. Pictures of NMuMG-cells are shown in Additional file 2: Figure S4. Scale bar = 0.02 mm. Proteins are colored red and nuclei were stained with DAPI (blue). Arrows indicate the location of the annotated protein in cells cultured in DMEM + rTGF-β1, 7d. (**D**) Relative luciferase expression (TOPFLASH/FOPFLASH) in F9-cells (left) and NMuMG-cells (right) after 7d or 13d of culture, in DMEM ± rEGF (50 ng/ml) or rTGF-β1 (2 ng/ml), respectively.

**Figure 5:**
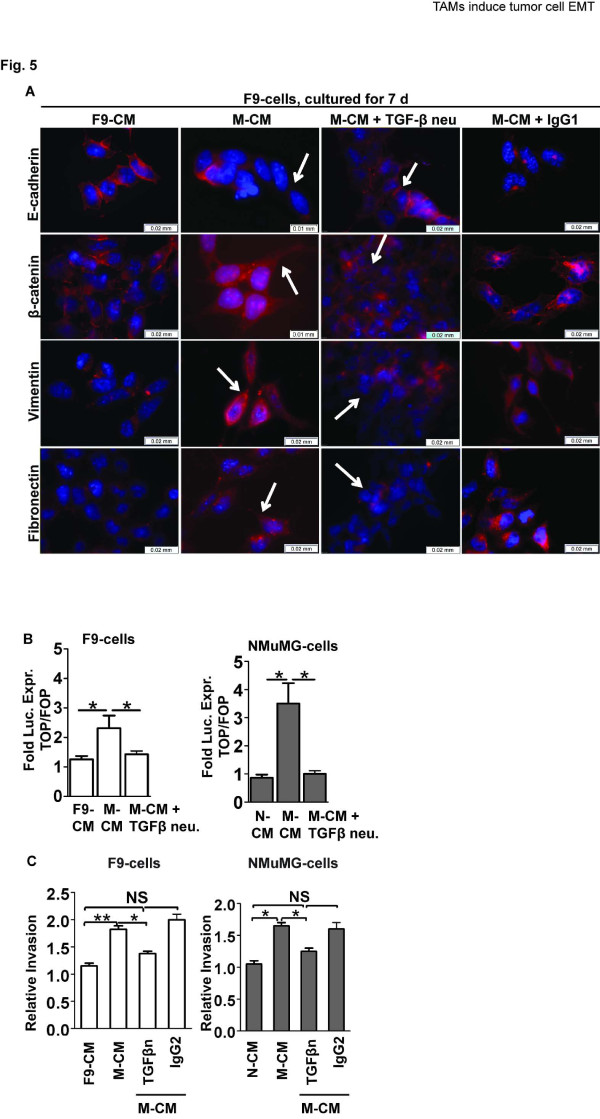
**M2 macrophages regulate EMT through paracrine TGF-β signaling**. (**A**) Immunofluorescence of E-cadherin, β-catenin, vimentin and fibronectin expression and cellular localization in F9-cells cultured in F9-CM, M-CM, M-CM neutralized for TGF-β and in M-CM plus an IgG_1 _control antibody for 7d. Pictures of NMuMG-cells are shown in Additional file 2: Figure S5. Scale bar = 0.02 mm. Proteins are colored red and nuclei were stained with DAPI (blue). Arrows indicate the location of the annotated protein in cells cultured in M-CM and M-CM + TGF-β neutralizing antibody for 7d. (**B**) Relative luciferase expression (TOPFLASH/FOPFLASH) in F9-cells (left) and NMuMG-cells (right) after 7d or 13d of culture in F9-CM, N-CM, M-CM or M-CM neutralized for TGF-β, respectively; n = 4-7; bar = means ± SEM. *P < 0.05, unpaired *t*-test. (**C**) Relative invasion by F9-cells (left) and NMuMG-cells (right) in response to F9-CM, N-CM, M-CM, M-CM neutralized for TGF-β (TGF-βn), or to M-CM plus an IgG_1 _control antibody (48h, 1% Matrigel); n = 3-8. Fold invasion was calculated relative to F9-CM or N-CM. *P < 0.05, **P < 0.005, unpaired *t*-test, bar = means ± SEM.

As the F9-and NMuMG cells never were in physical contact with macrophages we concluded that soluble factors present in the M-CM were responsible for induction and regulation of the observed trans-differentiation. To identify the specific signaling molecules involved in EMT induction, the F9-tumors were evaluated for gene expression of 4 macrophage derived factors previously associated to EMT and tumor cell invasion, namely *Wnt5a*, *Tgf-β1*, *Tgf-β2 *and *Egf *[16,29-34]. Whereas *Wnt5a *expression was too low for detection by q-PCR, decreased levels were found for *Egf *(~5-fold), *Tgf-β1 *(~ 2 fold) and *Tgf-β2 *(~7-fold) in clodrolip treated tumors relative to controls (Figure 4A, Additional file 2: Figure S3). Again, we used the five size matched, individual tumors from a single depletion study to address the correlative relationship between macrophage infiltration (*Csfr-1 *levels) and expression of the growth factors. Whereas *Egf *levels were down regulated in clodrolip treated tumors independently of *Csfr-1 *levels, expression of *Tgf-β1 *and *Tgf-β2 *correlated positively with *Csfr-1 *expression (Figure 4B). Macrophages are a main source of TGF-β1, whereas TGF-β2 to a large extent is secreted by tumor cells [51]. We therefore tested the cells ability to undergo EMT in response to recombinant EGF (rEGF, 50 ng/ml) and recombinant TGF-β1 (rTGF-β1, 2 ng/ml) in cell culture. Both cell lines responded to rEGF and rTGF-β stimuli by increased levels of pErk42/44 and phosphorylated tyrosine (data not shown). However, only rTGF-β1 induced mesenchymal trans-differentiation in both cell lines (Figure 4C, Additional file 2: Figure S4) which correlated with increased β-catenin/Tcf transcription activity (Figure 4D). To evaluate the importance of TGF-β in macrophage induced trans-differentiation, we neutralized TGF-β in M-CM prior to F9-and NMuMG-cell culturing (Figure 5A, Additional file 2: Figure S5 and data not shown). Whereas both cell lines underwent a mesenchymal transition when cultured in M-CM containing a control IgG_1 _antibody, the cells cultured in M-CM neutralized for TGF-β maintained an epithelial morphology and phenotype after 7 and 13 days of culture, respectively. Concordantly, TGF-β neutralization abolished M-CM mediated activation of the β-catenin/Tcf transcription complex (Figure 5B) and decreased the invasive properties of the cells (Figure 5C). Thus, we conclude that TGF-β is indispensable for induction of mesenchymal trans-differentiation by M2 macrophages in our cell lines.

### CD68^+ ^macrophage density correlates with a mesenchymal tumor cell phenotype, intraepithelial TGF-β levels and grade in non small cell lung cancer patients

**Figure 6:**
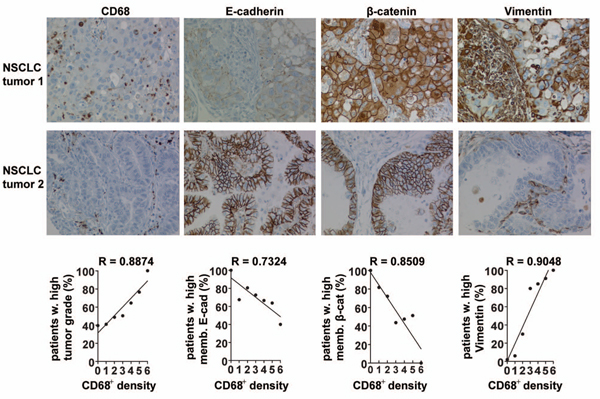
**Intratumoral CD68^+ ^macrophage density correlates with mesenchymal protein expression in NSCLC patient samples**. NSCLC tissue sections were stained for CD68, E-cadherin, β-catenin and vimentin. NSCLC tumor 1 is an adenocarcinoma of solid subtype, NSCLC tumor 2 is an adenocarcinoma of glandular subtype. NSCLC tumor 1 is a score 2 for CD68, score 1 for membranous E-cadherin, score 2 for membranous β-catenin, score 3 for cytoplasmic β-catenin and score 3 for vimentin. NSCLC tumor 2 is a score 1 for CD68, score 3 for membranous E-cadherin, score 3 for membranous β-catenin, score 1 for cytoplasmic β-catenin and score 0 for vimentin. Pictures were acquired at 20 × magnification. Graphs indicate correlations between intratumoral CD68^+ ^macrophage infiltration and tumor cell expression of the annotated EMT-associated markers. The regression coefficient R is indicated.

We have previously demonstrated a significant correlation between tumor cell expression of selected EMT-markers and relevant clinico-pathologic parameters of tumor progression in NSCLC patients [26]. In this study we used the same tissue microarray cohort to evaluate the relationship between intra-tumoral macrophage infiltration and EMT. Two independent researchers analyzed 491 NSCLC tissue samples of which 228 were adenocarcinomas (AC), 244 squamous cell carcinomas (SCC) and 19 adeno-squamous carcinomas (ASQ). Intra-tumoral macrophage density correlated significantly with tumor grade (Spearman correlation, SC > 0.2), but not with other clinico-pathologic parameters of tumor progression like pT, pN, pM or tumor size (Table 1). The TMAs were further analyzed for tumor cell expression of EMT-associated markers and TGF- β (Table 2 and Figure 6). Membranous E-cadherin and membranous β-catenin were chosen as epithelial markers, whereas vimentin, periostin and cytoplasmic β-catenin were chosen as mesenchymal markers. Both evaluations established a moderate, positive correlation between intratumoral CD68^+ ^macrophage density, mesenchymal marker expression and TGF-β1 levels in tumor cells (SC > 0.2). Moreover, CD68^+ ^macrophage density correlated negatively with membranous β-catenin in both evaluations (SC > -0.2). E-cadherin likewise showed a modest negative correlation with CD68^+ ^macrophage density in evaluation 1 (SC > -0.2), however, evaluation 2 could not confirm this correlation. Finally, expression of EMT markers correlated positively with tumor grade (SC < 0.2). This data suggests that the parameter tumor grade is most closely related to increased amounts of intratumoral macrophages, intraepithelial TGF-β levels and expression of EMT markers.

**Table 1:** Clinico-pathologic parameters and correlation with CD68^+ ^macrophage tumor infiltration

Tumors	**No**.	%		CD68^+ ^intra-tumoral TAMs	
n = 491				Evaluation 1 Evaluation 2	
**Histotype**					
AC	228	46.4			
SCC	244	49.7			
ASQ	19	3.9			

**pT**					
T1	100	20.4	SC	0.000	0.008
T2	268	54.6	*p*	0.993	0.859
T3	74	15.1			
T4					
**pN**					
N0	257	52.3	SC	-0.022	-0.072
N1	147	29.9	*p*	0.706	0.116
N2	77	15.7			
N3	10	2.0			
**pM**					
M0	448	91.2	SC	-0.117	-0.006
M1	43	8.8	*p*	0.082	0.161
**Grade**					
G1	28	5.7	SC	0.227	0.025
G2	253	51.1	*p*	≤ 0.001*	≤ 0.001*
G3	210	42.8			
**Size**					
≤ 3.7 cm	246	50.1	SC	0.072	0.080
> 3.7 cm	245	49.9	*p*	0.114	0.079

*for G1 and G2 vs. G3

*Abbreviations: AC *Adenocarcinoma, *SCC *Squamous Cell Carcinoma, *ASQ *Adeno-squamous Carcinoma

*p*-values and Spearman correlation values (SC) are indicated. Statistical analysis was done with SPSS 16.0

**Table 2:** Correlation of EMT-associated protein markers and TGF-β with CD68^+ ^macrophage tumor infiltration

Proteins		CD68^+ ^intratumoral TAMs		Grade
		Evaluation 1 Evaluation 2		1-3
**E-cadherin membrane**	SC	-0.001	-0.073	-0.104
	*p*	*0.035*	0.111	*0.021*

**β-Catenin membrane**	SC	-0.174	-0.175	-0.190
	*p*	*≤ 0.001*	*≤ 0.001*	*≤ 0.001*

**β-Catenin cytoplasm**	SC	0.223	0.231	0.163
	*p*	*≤ 0.001*	*≤ 0.001*	*≤ 0.001*

**Vimentin cytoplasm**	SC	0.225	0.485	0.178
	*p*	*≤ 0.001*	*≤ 0.001*	*≤ 0.001*

**Periostin cytoplasm**	SC	0.177	0.215	0.112
	*p*	*≤ 0.001*	*≤ 0.001*	*0.008*

**TGF-β intraepithelial**	*SC*	0.230	0.184	*0.254*
	*p*	*≤ 0.001*	*≤ 0.001*	*≤ 0.001*

*p*-values and Spearman correlation values (SC) are indicated. Statistical analysis was done with SPSS 16.0. Tumors, n = 491

## Discussion

The recognition that disease progression is highly influenced by the tumor microenvironment has lead to the concept that cancer management may be improved by therapeutic targeting of the tumor stroma [11,52,53]. We and others have previously demonstrated the potential therapeutic value of macrophages by showing that their depletion or inhibition of their recruitment leads to reduced angiogenesis and tumor growth, increased tumor necrosis and reduced metastasis in tumor bearing mice [11,51]. Moreover, M1 macrophages have been shown to regulate EMT at the invasive front through paracrine TNF-α signaling and Snail stabilization, linking tumor inflammation to EMT and metastasis [20,42]. In this study, we show that the role of tumor associated macrophages in tumor cell EMT extends beyond the invasive front.

Gene expression analysis of control and clodrolip treated tumors revealed a positive correlation between macrophage infiltration and mesenchymal marker expression in whole F9-tumors. Moreover, immunohistochemical analysis of the tumors confirmed a positive correlation between macrophage and mesenchymal tumor cell density, suggesting that TAMs modulate the phenotype of tumor cells located in the neighboring microenvironment. This trend was confirmed *in vitro *using a conditioned medium approach to facilitate trans-differentiation in F9-and NMuMG-cells. As assayed by immunofluorescence, the mesenchymal phenotype was reversible in F9-cells upon M-CM removal *in vitro*. Fibroblastoid epithelial cancer cells can re-acquire epithelial traits by undergoing mesenchymal-epithelial transition (MET). MET has been assigned with a hypothetical role in metastatic colony formation where plastic tumor cells, in response to micro-environmental changes, reacquire epithelial traits [19,54]. That the M-CM induced mesenchymal phenotype was reversible *in vitro*, suggests that TAMs incite tumor cell plasticity and contribute to tumor heterogeneity through regulation of EMT/MET. The occurrence of EMT in tumors may therefore be transient and highly dependent on the local microenvironment.

The β-catenin pathway is an important mediator of EMT [19,34]. Although immunoblotting analysis of whole tumor tissues failed to establish a noticeable difference in the levels of active β-catenin in the two F9-tumor groups, *in vitro *systems utilizing the TOPFLASH/FOPFLASH reporter system confirmed a significant increase in transcriptional activity in both F9-and NMuMG cells upon long term M-CM culturing. Together with the immunohistochemical data, which indicated that macrophages mainly cluster with cells expressing biochemical EMT markers locally in the tumor, we conclude that the levels of active β-catenin in F9-tumors were too low to be detected by western blotting. Therefore, based on the *in vitro *data we suggest that M2 macrophages signal EMT in part through activation of the β-catenin pathways.

In a candidate based screen we identified TAM-derived TGF-β as the main cytokine inducing EMT in our assays. TGF-β is a well characterized regulator of EMT and it can induce epithelial trans-differentiation through various pathways. On one hand, TGF-β induces EMT through activation of various intrinsic pathways, e.g. AKT, SMAD and β-catenin [19,29-32]. Conversely, it stimulates the production of MMPs which are important stroma-derived inducers of tumor cell EMT [25,35]. Although being a strong mediator of EMT, TGF-β often signals in conjunction with other cytokines such as TNF-α, Wnt and EGF [40,55,56]. We were therefore surprised by the finding that neutralization of TGF-β in M-CM was sufficient to abrogate the invasive, mesenchymal cell phenotype. Although we cannot exclude synergistic effects between TGF-β and other macrophage-derived cytokines, we conclude that TGF-β is indispensable for the induction of EMT in our cell systems. All together, the data suggest a model in which M2 polarized macrophages regulate EMT through TGF-β signaling and consecutive activation of the β-catenin pathway. This finding challenges the previous finding of M1 macrophages signaling EMT at the invasive front through paracrine TNF-α signaling and consecutive SNAIL activation [21,42]. Our findings suggest that the signaling pathways through which macrophages induce EMT is highly context dependent.

An important aspect of this study was to address the clinical relevance of TAM regulated EMT. For this purpose we analyzed tumor samples of NSCLC patients in which we had previously established significant correlations between tumor cell expression of EMT markers and disease outcome [26]. In the current study, we evaluated the correlation between intra-tumoral CD68^+ ^macrophage density and EMT profile. In concordance with the murine data, the NSCLC study revealed a positive, significant correlation between CD68^+ ^macrophage density, intraepithelial TGF-β levels and expression of EMT markers in adjacent tumor cells. Moreover, intratumoral CD68^+ ^density, intraepithelial TGF-β1 levels and EMT tumor profile correlated with high tumor grade. Although TAMs and tumor cell EMT generally are associated with metastasis, we did not obtain evidence for such correlation (Table 1) and it is important to note that the metastatic process is complex and not exclusively depending on these two factors.

## Conclusions

Collectively, the data suggest a model in which TAMs induce EMT in intratumoral epithelial cells through paracrine TGF-β signaling and consecutive activation of the β-catenin pathway. As macrophage infiltration, TGF-β1 expression and pronounced EMT tumor phenotypes correlate with increased grade in NSCLC patients, we propose that TAMs contribute to tumor progression by inducing mesenchymal trans-differentiation in local clusters in tumors and thereby contribute to tumor heterogeneity and grade. As EMT is associated with both drug resistance and patient relapse it is attractive to speculate that therapeutic targeting of tumor associated macrophages could improve disease outcome.

## Competing interests

The authors declare that they have no competing interests.

## Authors' contributions

AKB designed and carried out the experiments, participated in the evaluation of the clinical data and drafted the manuscript. VT participated in the evaluation of the clinical data and helped to draft the manuscript, SK participated in the design of the study and critically revised the manuscript, AS participated in the evaluation of the clinical data and helped to draft and revise the manuscript. RAS conceived the study, participated in its design and coordination and helped to write the manuscript. All authors read and approved the final manuscript.

## Pre-publication history

The pre-publication history for this paper can be accessed here:

http://www.biomedcentral.com/1471-2407/12/35/prepub

## Supplementary Material

Additional file 1 **Table S1**. Q-PCR primer sequences. Click here for file

Additional file 2 **Figure S1**. Immunofluorescence analysis of EMT-associated marker expression in NMuMG cells. **Figure S2**. Protein expression analysis of F9-and NMuMG cells cultured in F9/N-CM and M-CM. **Figure S3**. *Wnt5a *gene expression in F9 teratocarcinomas. **Figure S4**. rTGF-β induced EMT correlates with activation of the β-catenin pathway. **Figure S5**. Neutralization of TGF-β abrogates M-CM induced EMT in NMuMG cells *in vitro*. Click here for file
