# Supplementary material for: Intratumoral macrophages contribute to epithelial-mesenchymal transition in solid tumors
Source: BMC Cancer. 2012 Jan 24;12:35. doi: 10.1186/1471-2407-12-35 (PMC3314544; doi:10.1186/1471-2407-12-35)
Supplement: Additional file 1 — Table S1. Q-PCR primer sequences. [file 1471-2407-12-35-S1.PDF]

Supplementary Table S1. Q-PCR primer sequences

**Primer name**	**Primer Sequence**	**Annealing Temp/°C**
β-actin forward	5'- CTC TTC CAG CCT TCC TTC CTG-3'	57
β-actin reverse	5'- GAA GCA TTT GCG GTG GAC GAT-3'	57
CD68 forward	5`- CTT ACC TTT GGA TTC AAA CAG GAC C-3`	59
CD68 reverse	5`- CCA TGA ATG TCC ACT GTG CTG C-3`	59
CSFR-1 forward	5`- CTA GGA CAA AGC AAG CAG CTC C-3`	56
CSFR-1 reverse	5`- CTG GTA CTT CGG CTT CTG CTT G-3`	56
E-cadherin for.	5`- GCA GGTCTC CTC ATG GCT TTG C-3`	57
E-cadherin rev.	5`- CCT TCA AAT CTC ACT CTG CCC AGG-3`	57
EGF forward	5'-GCG AGG ATG TCA ATG AAT GTG C-3'	57
EGF reverse	5'- GTT CGT GAC ATT GTT TCC CAT C-3'	57
GAPDH forward	5`- GAC ATT GTT GCC ATC AACGAC C-3'	58
GAPDH reverse	5`- CCC GTT GAT GAC CAG CTTCC-3'	58
N-cadherin for.	5`- CTT GAG AGC ACA TGC AGT GGA C-3`	61
N-cadherin rev.	5`- CGT CAT CAC ATA CGT CCC AGG-3`	61
Snail forward	5`-GAC GCG TGT GTG GAG TTC ACC-3'	60
Snail reverse	5`-GAT GAG GGT GGG CAG CGA AG-3'	60
TGF-β1 forward	5'-GGA ACT CTA CCA GAA ATA TAG C-3'	57
TGF-β1 reverse	5'-CCT GTA TTC CGT CTC CTT G-3'	57
TGF-β2 forward	5'-CCG AGC AGC GGA TTG AAC TG-3'	57
TGF-β2 reverse	5'-GCG TCT GTC ACG TCG AAG G-3'	57
Twist forward	5`- GCA AGC CAG GAC CCA CCT G-3'	63
Twist reverse	5`- GAT GTG AAC CTG GGT CTC TGT C-3'	63
Vimentin for.	5`-GTC CAA GTT TGC TGA CCT CTC TG-3`	58
Vimentin rev.	5`- CTC CAG GGA CTC GTT AGT GCC-3`	58
Wnt5a forward	5'-GAA GCC CAT TGG AAT ATT AAG C-3'	56
Wnt5a reverse	5'-GTT ATT CAT ACC TAG AGA CCA C-3'	56
