# Supplementary material for: Intratumoral macrophages contribute to epithelial-mesenchymal transition in solid tumors
Source: BMC Cancer. 2012 Jan 24;12:35. doi: 10.1186/1471-2407-12-35 (PMC3314544; doi:10.1186/1471-2407-12-35)
Supplement: Additional file 2 — Figure S1. Immunofluorescence analysis of EMT-associated marker expression in NMuMG cells. Figure S2. Protein expression analysis of F9-and NMuMG cells cultured in F9/N-CM and M-CM. Figure S3. Wnt5a gene expression in F9 teratocarcinomas. Figure S4. rTGF-β induced EMT correlates with activation of the β-catenin pathway. Figure S5. Neutralization of TGF-β abrogates M-CM induced EMT in NMuMG cells in vitro. [file 1471-2407-12-35-S2.PDF]

Supplementary figure S1

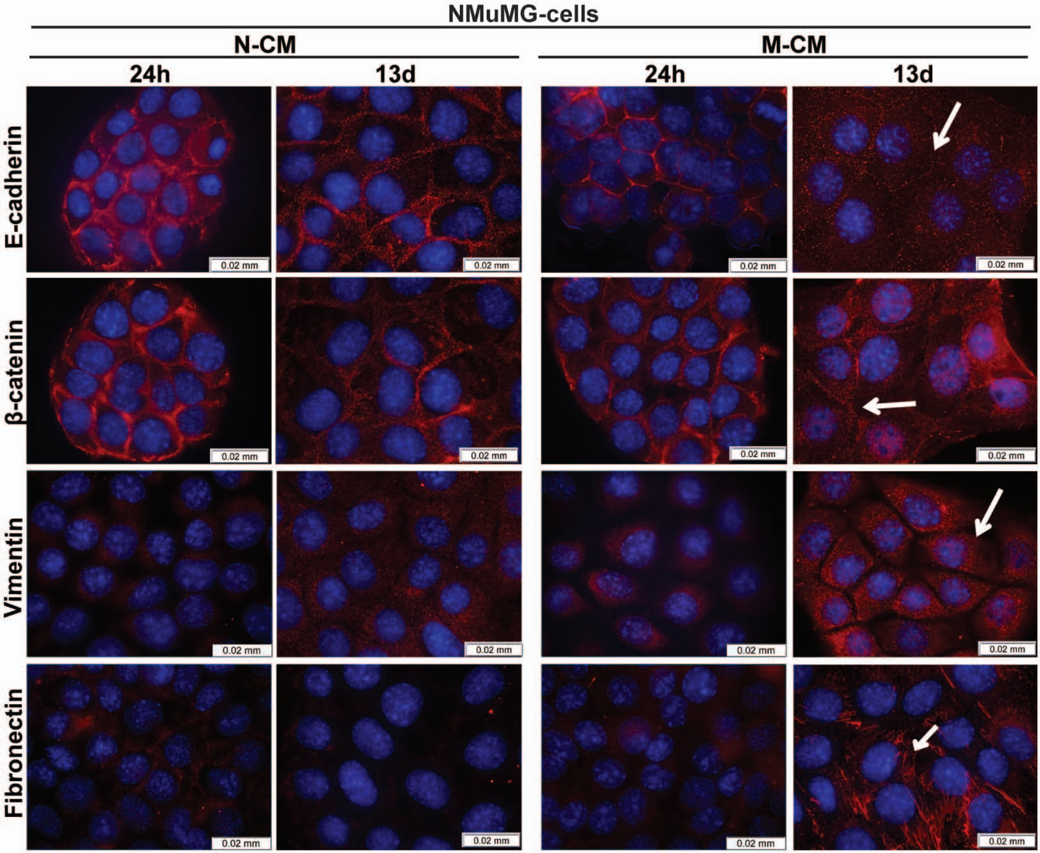

Fig. S1: Immunofluorescence analysis of EMT-associated marker expression in NMuMG-cells. Immunofluorescence analysis of E-cadherin, β-catenin, vimentin and fibronectin (all in red) expression and localization in NMuMG-cells cultured in N -CM or M-CM for 24 h or 13 d. Scale bar = 0.02 mm. Nuclei were stained with DAPI. Arrows indicate the location of the annotated protein in cells cultured in M-CM, 13d.

Supplementary figure S2

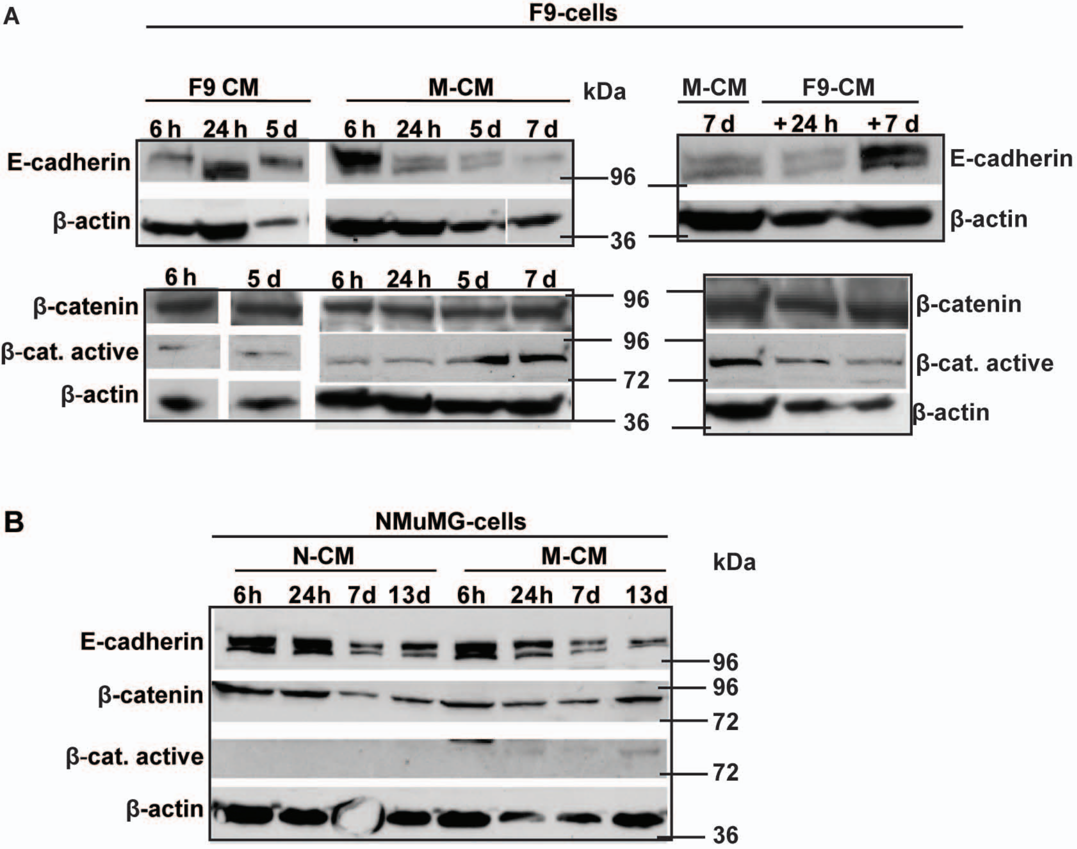

Fig. S2: Protein expression analysis of F9- and NMuMG- cells cultured in F9/ N-CM and M-CM. A: Western blotting analysis of E-cadherin, β- catenin and active β-catenin expression in F9-cells cultured in F9-CM or M-CM at annotated timepoints. **B:** Western blotting analysis of E- cadherin, β- catenin and active β- catenin expression in NMuMG-cells cultured in N-CM or M-CM at annotated timepoints.

Supplementary figure S3

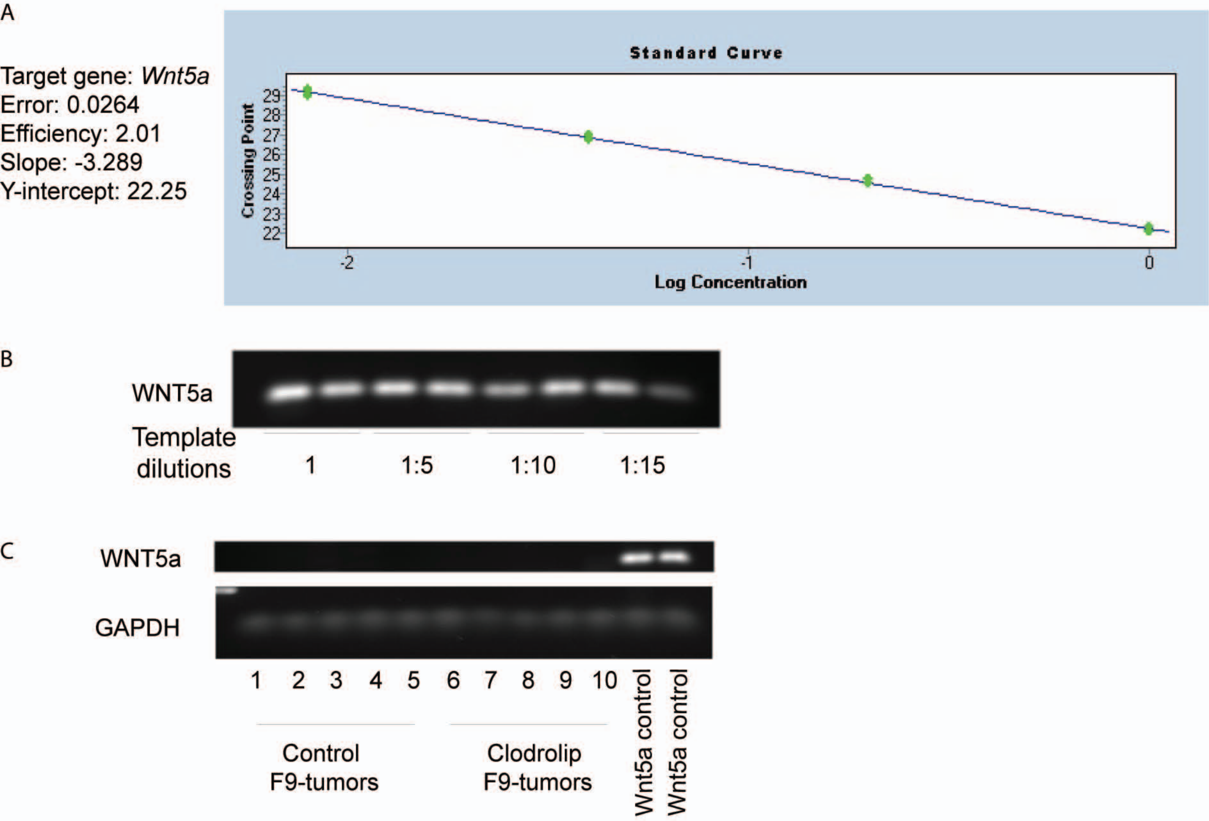

Fig. S3: ***Wnt5a* gene expression in F9 teratocarcinomas. A:** Standard curve generated from 4 dilution of *wnt5a* expressing cDNA template.**B:** PCR products from a *Wnt5a* overexpressing template, evaluated on a 1.5% agarose gel (dilutions: 1, 1:5, 1:10 and 1:15).**C:** PCR products obtained from the reactions using template from 5 control F9-tumors, 5 clodrolip F9-tumors and 2 *Wnt5A* expressing control templates

Supplementary figure S4

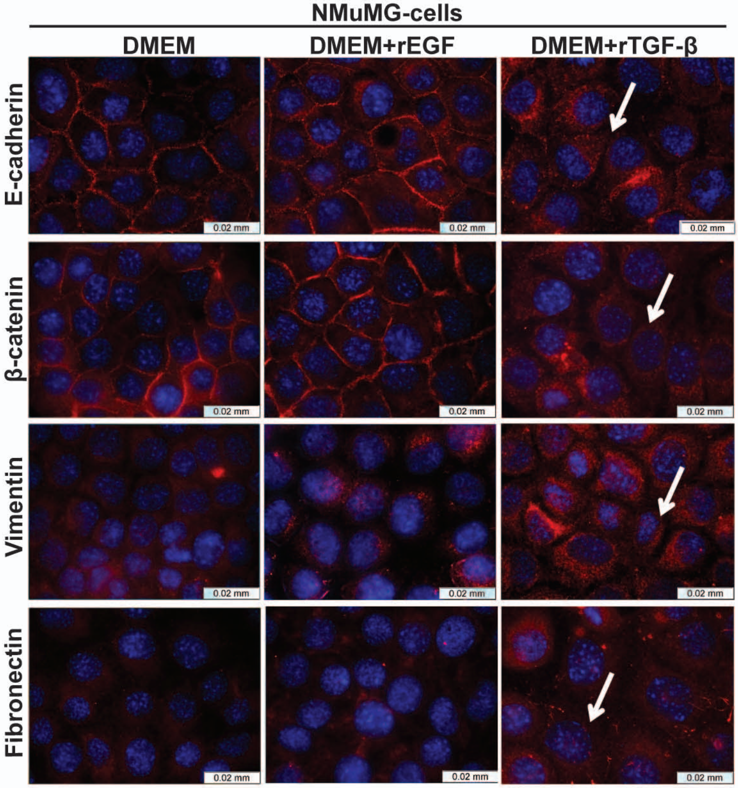

Fig. S4: rTGF-β1 induced EMT correlates with activation of the β-catenin pathway *in vitro*. Immunofluorescence analysis of E - cadherin, β - catenin, vimentin and fibronectin (all in red) expression in NMuMG-cells cultured in DMEM+/- rEGF (50 ng/ml) and DMEM+/- rTGF-β1 (2 ng/ml) for 13d. Scale bar = 0.02 mm. Nuclei were stained with DAPI. Arrows indicate location of the annotated protein in cell cultured in M-CM, 13d.

Supplementary figure S5

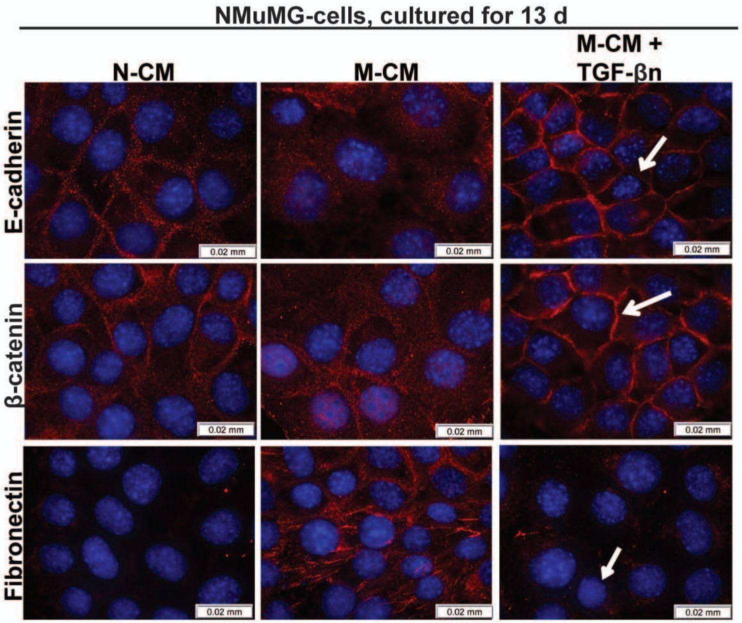

Fig. S5: Neutralization of TGF- β abrogates M- CM induced EMT in NMuMG-cells *in vitro*. Immunofluorescence analysis of E-cadherin, β- catenin and fibronectin (all in red) expression in NMuMG - cells cultured for 13 d in N - CM, M - CM or M - CM neutralized for TGF-β. Scale bar = 0.02 mm. Nuclei were stained with DAPI. Arrows indicate location of the annotated proteins in cells cultured in M - CM + TGF - β neutralizing antibody.
